# Timing Is Critical for Effective Glucocorticoid Receptor Mediated Repression of the cAMP-Induced CRH Gene

**DOI:** 10.1371/journal.pone.0004327

**Published:** 2009-01-29

**Authors:** Siem van der Laan, E. Ronald de Kloet, Onno C. Meijer

**Affiliations:** Division of Medical Pharmacology, Leiden/Amsterdam Center for Drug Research and Leiden University Medical Center, Leiden, The Netherlands; James Cook University, Australia

## Abstract

Glucocorticoid negative feedback of the hypothalamus-pituitary-adrenal axis is mediated in part by direct repression of gene transcription in glucocorticoid receptor (GR) expressing cells. We have investigated the cross talk between the two main signaling pathways involved in activation and repression of corticotrophin releasing hormone (CRH) mRNA expression: cyclic AMP (cAMP) and GR. We report that in the At-T20 cell-line the glucocorticoid-mediated repression of the cAMP-induced human CRH proximal promoter activity depends on the relative timing of activation of both signaling pathways. Activation of the GR prior to or in conjunction with cAMP signaling results in an effective repression of the cAMP-induced transcription of the CRH gene. In contrast, activation of the GR 10 minutes after onset of cAMP treatment, results in a significant loss of GR-mediated repression. In addition, translocation of ligand-activated GR to the nucleus was found as early as 10 minutes after glucocorticoid treatment. Interestingly, while both signaling cascades counteract each other on the CRH proximal promoter, they synergize on a synthetic promoter containing ‘positive’ response elements. Since the order of activation of both signaling pathways may vary considerably *in vivo*, we conclude that a critical time-window exists for effective repression of the CRH gene by glucocorticoids.

## Introduction

Corticotropin releasing hormone (CRH) is a pivotal signaling molecule in the regulation of the stress response. This neuropeptide is expressed at high levels in the hypothalamus, from where it coordinates the hormonal and autonomic response to stress, and the central nucleus of the amygdala, where it plays a crucial role in regulating anxiety. Regulation of the expression of CRH is therefore thought to be physiologically important in relation to coping with stress. CRH gene regulation is a complex process that involves multiple activating and repressing transcription factors [1]. Among the often studied factors that can regulate CRH expression are glucocorticoid hormones [2], which in a cell-dependent manner either repress of stimulate the CRH gene. As such, the CRH promoter can be considered a model gene for cell-specific negative regulation of gene expression via glucocorticoids.

Cross-talk of intracellular signaling pathways is central to many neuroendocrine control systems [3], [4]. The expression and/or secretion of the two main neuroendocrine secretagogues of the hypothalamus-pituitary-adrenal axis (HPA axis) are both stimulated by cAMP and suppressed by glucocorticoids, the end-product of the HPA axis: hypothalamic CRH, as well as adrenocorticotrophic hormone (ACTH) from anterior pituitary corticotrophs [5]–[8]. At the molecular level, these signals are represented by protein kinase A (PKA), the transcription factor cAMP element-binding protein (CREB), and the GR, respectively.

**Figure 1:**
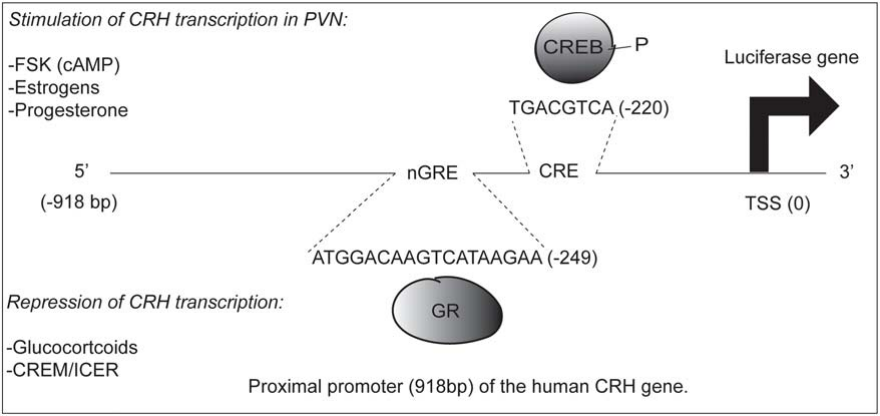
Simplified representation of the hCRH-luc promoter and known response elements. Schematic representation of the composite hCRH proximal promoter, as present in the reporter construct. Although only the known nGRE and CRE have been indicated, many response elements have been identified within the used reporter construct, such as a functional estrogen response element half site [28], and several putative AP1 sites [14], [25]. In addition, some of the listed factors act on sequences that are not present in reporter construct [29].

The proximal promoter of the human corticotrophin releasing hormone (hCRH) gene contains a canonical, functional cAMP response element (CRE) and a negative glucocorticoid receptor response element (nGRE) (fig. 1). Induction of the hCRH gene by the PKA pathway is mediated by phosphorylation of CREB at serine residue 133 [9], [10]. *In vivo*, Wölfl *et al.* showed that binding of CREB to the canonical CRE located at the nucleotide position −224 (upstream of exon 1) was specifically induced after activation of the PKA pathway with forskolin [11]. Additionally, Kovacs *et al.* demonstrated that in the hypothalamic parvocellular neurons of rodents subjected to ether stress, CREB phosphorylation was induced in a time course that parallels the increase of CRH heteronuclear RNA levels [12].

*In vitro,* the At-T20 cell-line is a well-established model system for studying glucocorticoid-induced repression of the hCRH proximal promoter. Nested deletions and site-specific point mutations of the CRE located at nucleotide −224 resulted in a significant loss of induction by cAMP, demonstrating that CREB binding is necessary for the stimulation of the gene [13]. In parallel, electrophoretic mobility shift assays (EMSA) identified a GR-binding site at position nt −249 that was indispensable for GR-mediated repression of the cAMP-induced promoter. Internal deletion of the entire nGRE and specific point mutations resulted in a loss of repression by the ligand-activated GR, indicating that DNA binding is essential for the glucocorticoid-induced repression [14]. Of note: while we have taken this nGRE-mode as working model, a separate series of experiments did not find evidence for direct GR binding to the CRH promoter, but rather suggested direct CREB-GR interactions as the cause of GR-mediated reression [15].

The nGRE in the hCRH promoter is separated by as few as 25 bp with the canonical CRE, a distance that clearly permits functional interactions at the promoter [16]. Since, *in vivo* the order of activation of the cAMP and glucocorticoid signaling pathways may vary considerably, and this is known to affect responses at the level of neuroendocrine secretion [17], we tested the hypothesis that effective repression of the cAMP-induced hCRH proximal promoter depends on the relative timing of GR activation in the At-T20 cell-line.

## Results

### Dexamethasone pre- or simultaneous co-treatment with FSK

**Figure 2:**
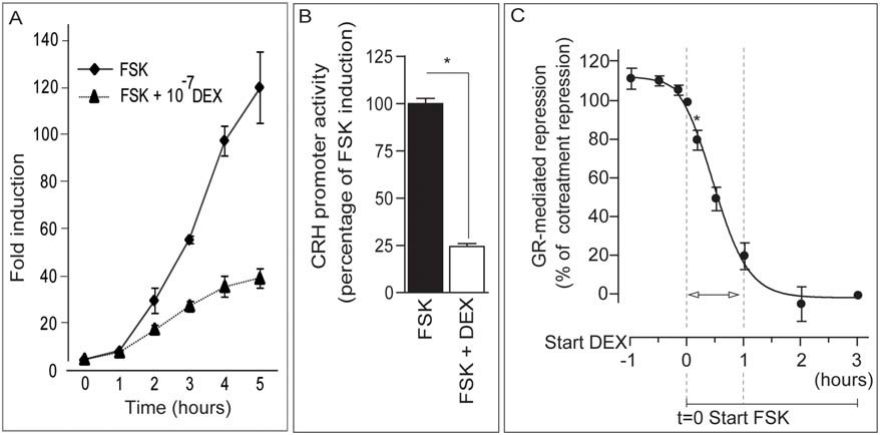
Relative timing of DEX and FSK treatment determines efficacy of GR-dependent repression of CRH-promoter activity. (2A) FSK-stimulation progressively induces the CRH-promoter activity in the Att20 cells over time. Co-treatment with DEX resulted in a repressed CRH-activity. 2B) CRH-promoter activity expressed as percentage of maximal induction after 3 hours forskolin (FSK) treatment (filled bar). Simultaneous co-treatment with DEX (open bar) resulted in a strong repression of the CRH-promoter activity. (2C) The repression induced by DEX in the co-treatment group was set at 100%. All groups were treated for three hours with FSK. Different time of onset of the DEX treatment relative to the FSK treatment results in a significant loss of repression when DEX treatment is started 10 minutes after FSK treatment (*). FSK treatment leads to a progressive increase in CRH-luc promoter activity over a period of at least 5 hours (inset). Values represent group averages ± SD.

FSK treatment led to a robust and progressive stimulation of the CRH-promoter activity that was evident for luciferase induction from 1 hour to at least 5 hours (fig. 2A). In line with previous reports [14], [18], simultaneous DEX co-treatment strongly suppressed the FSK-induced stimulation of the hCRH-promoter activity (fig. 2A). DEX co-treatment resulted in up to 75% repression of the FSK-induced promoter activity after 3 hours treatment (fig. 2B). To test our hypothesis that the order of activation of both signaling cascades affects the level of GR-mediated repression, we initiated the DEX treatment at different time points prior to or after initiation of the 3-hours FSK treatment (fig. 2C). We compared the DEX-induced repression in the different groups to the simultaneous co-treatment group, which was set at 100% repression. Two hours of DEX pre-treatment resulted in a significantly increased repression, suggesting that a slower mechanism requiring *de novo* protein synthesis is responsible for the additional repression (data not shown). Activation of the GR up to one hour prior to FSK treatment did not affect the levels of repression (fig 2C, first three time points). Of note, DEX treatment alone (0.1 μM) did not suppress the basal activity of the CRH-promoter, indicating that basal CRH drive is not governed by CREB/CRE dependent mechanisms (data not shown).

### Dexamethasone treatment applied after FSK

When DEX was applied after forskolin stimulation of the CRH promoter, the time-window separating both treatments was of great consequence for the level of repression (fig. 2C). A 10 minutes delay in the onset of DEX treatment relative to the FSK treatment resulted in a loss of 20% repression. Strikingly, a 30 minutes delay resulted in a 50% loss of GR-mediated repression, indicating the importance of the relative time of treatment. Clearly, the reduced time of DEX exposure is not proportional to the loss of GR-mediated repression pointing to a ‘GR resistance’. Because FSK treatment induces a progressive increase of the CRH-luc promoter activity over a period of at least 5 hours (fig. 2A) we assume that FSK-induces binding of CREB to the promoter over that period. However, the first hour following FSK treatment is critical for the GR to mediate effective repression.

**Figure 3:**
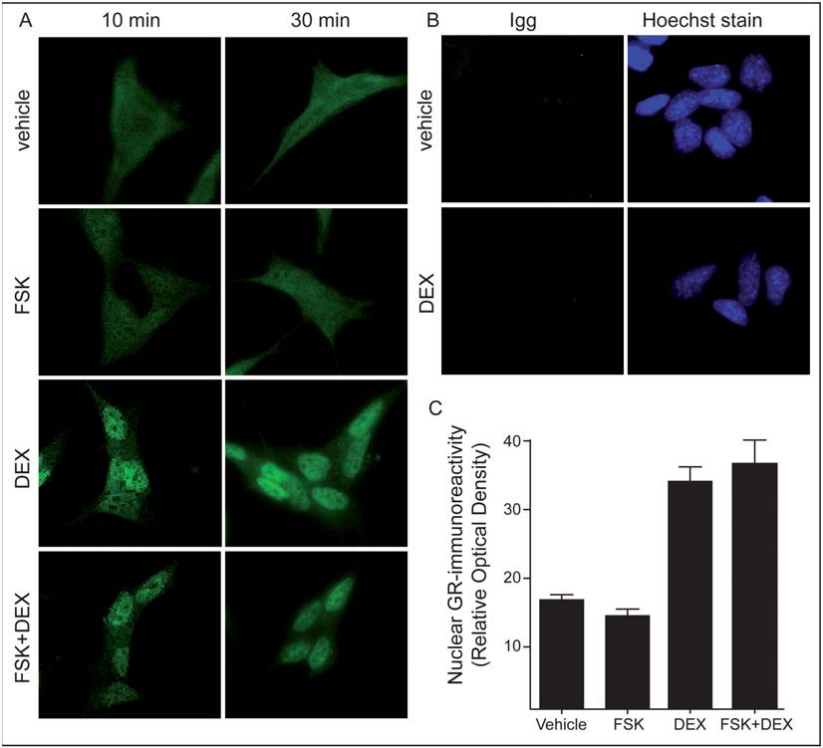
Translocation of the GR occurs within 10 minutes after treatment. (3A) Time course of GR-ir in different treatment groups. DEX alone and FSK + DEX co-treatment, but not FSK alone show nuclear GR staining after 10 minutes treatment. (3B) Control IgG staining show specificity of the GR-specific antibody. (3C) Nuclear quantification of GR-ir after 10 minutes treatment. Values represent average ±SEM.

To assess whether FSK treatment alters the translocation properties of the GR to the nucleus, we quantified GR-immunoreactivity in the different conditions. The data show that DEX treatment induces maximal nuclear GR-immunoreactivity (GR-ir) as early as 10 minutes after treatment (figure 3). No difference in nuclear GR-ir was observed between the 10 and 30 minutes DEX treatment groups, suggesting that the ‘GR resistance’ is not due to delayed translocation to the nucleus (fig. 3A). In addition, FSK treatment did not influence translocation dynamics of the GR although it is known that PKA activation can modulate the steroid sensitivity by enhancing DNA binding properties of GR [19]. In sum, the translocation data provide evidence that GR should be capable of modulating gene transcription as early as 10 minutes after treatment and that FSK treatment does not interfere with translocation related mechanisms.

### Promoter specificity

**Figure 4:**
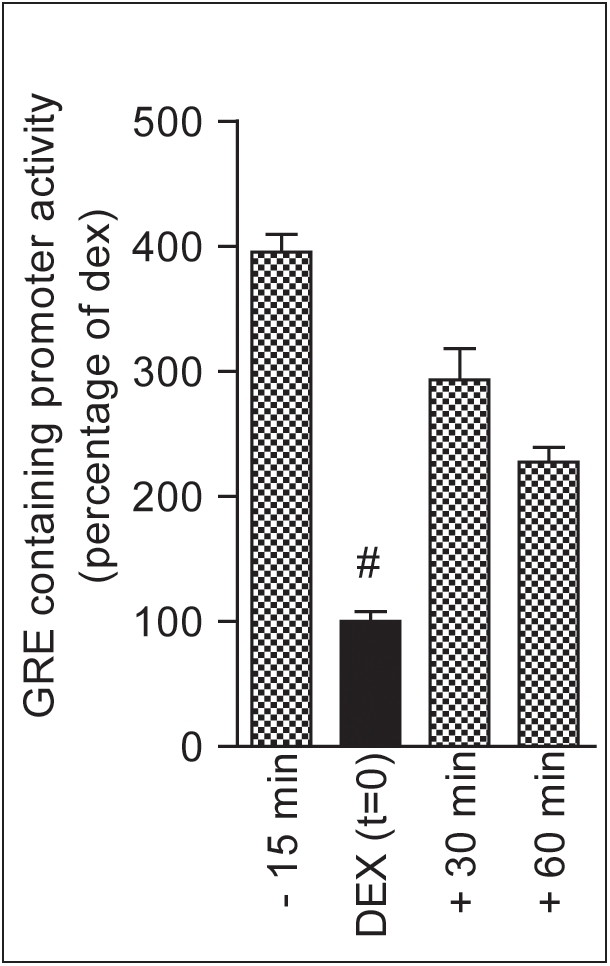
The FSK induced GR resistance is specific for the CRH promoter. TAT3-luc (GRE-containing promoter) activity is expressed as percentage of maximal induction after 4 hours DEX treatment (filled bar; t = 0). All groups (hatched bars) were treated for 4 hours with DEX and only the time of onset of FSK treatment was different. Forskolin treatment strongly enhanced the transcriptional rate of GR at all time points (# indicates significantly different from DEX group with p<0.05). Pre-treatment with FSK resulted in the highest potentiation of the GR transcriptional rate.

Posttranslational modification such as phosphorylation is known to affect DNA binding properties, transcriptional activation and stability of numerous nuclear receptors including GR [19]. Although translocation to the nucleus was not affected by FSK treatment, we tested whether FSK influenced the transcriptional activity of the GR in these cells. We measured the effect of FSK and DEX co-treatment on a positively regulated promoter (a synthetic GRE-containing promoter; TAT3-luc; [20]). FSK co-treatment synergistically induced transcription on an exclusively GRE-containing promoter compared to DEX treatment alone (fig. 4). FSK treatment prior DEX treatment resulted in an increased transcriptional activity of the GR. Likewise, the longer the time of FSK co-treatment the higher the transcriptional activity of the GR.

## Discussion

The current data demonstrate that time-dependent interactions between GR and cAMP/CREB can occur at the level of the CRH gene, where these factors seem to functionally compete for the same promoter. While similar interactions have been described for the secretion of ACTH from the pituitary [17], we now show that cAMP induced ‘GR-resistance’ occurs at the level of a single promoter, and that it is not a global cellular phenomenon, but gene-specific.

Using the nGRE that was reported to be functional in these cells as a working model [14], the observed ‘primacy’ effect for transcription factor action at the CRH promoter may be due to the close proximity of the two response elements involved. The spacing of the elements is such that it is unlikely that both GR and CREB may bind simultaneously in an independent manner [16]. Sterical hindrance at the promoter due to the formation of larger protein complexes may be responsible for the importance of timing of stimuli. Alternatively, in view of the dynamic nature of transcription factor-DNA interactions, CREB-mediated chromatin remodeling events that disfavor GR-binding may account for the decreased GR efficacy observed after prior cAMP elevations.

Interestingly, the analogous dependence of timing of both cAMP and GR that exists for ACTH secretion [17], which obviously is not linked to the activity of the exogenous reporter plasmid, suggests that the phenomenon of time-dependence occurs at multiple genes. Any gene regulated in a parallel manner will allow better hypotheses as to the mechanism that is responsible for the time dependent effects. POMC and CRH seem to depend on the same coregulator molecule, namely SRC-1 [18], [21]. In this respect it would be of great interest to also study negative regulation of the endogenous POMC gene in these cells under similar conditions as were used for the CRH reporter construct.

Although numerous studies were devoted to understanding the regulation of CRH gene expression in the paraventricular nucleus of the hypothalamus, it is still a topic of debate whether the activated-GR directly acts on the promoter region of the gene or that different mechanisms are responsible for the repression of CRH gene after stress. Bali *et al.* convincingly demonstrated in organotypic slice cultures that the GR directly acts on the paraventricular neurons to repress FSK-induced activity. However, the molecular mechanisms underlying this GR-mediated repression are still unknown. Guardiola-Diaz *et al.* suggested in 1996 that glucocorticoid repression occurs via interactions between the GR and the cAMP-responsive element-binding proteins [15], rather than via direct DNA binding of GR. In contrast, Dorin *et al.* provided evidence, also in the same cell line as used in present study, that the nGRE in the promoter is essential for repression by glucocorticoids [13]. It would certainly be of interest to study whether CREB phosphorylation status changes as a consequence of GR activation at different time points, and test the hypothesis that it is inversely related with the extent of GR repression. However, while CREB-driven transcription is repressed by glucocorticoids on the composite hCRH promoter, it is unaffected on a 5×CRE-containing promoter [18]. With the possible caveat that the 5×CRE may be not allow detection of subtle changes in CREB function, these data point to gene/promoter specificity of any direct CREB-GR interactions.

On the other hand, FSK-induced PKA can modulate glucocorticoid signaling both on the composite hCRH and the exclusively 3×GRE-containing promoters. Therefore, PKA activation can determine the transcriptional outcome at glucocorticoid target genes, independent of the presence of CREs in the promoter. We postulate that there is no cross-talk between the GR and CREB off the DNA but that PKA activation modulates GR-mediated transcription by changing e.g the phosphorylation status of coregulator proteins. Chromatin immunoprecipitation assays should be used to test the interactions between GR and (phospho-)CREB at the CRH promoter, and demonstrate lack of direct GR-CREB interactions at non-composite GRE-containing promoters.

An unexplained phenomenon is that, in contrast to the situation in PVN, glucocorticoids induce, rather than repress, CRH gene expression in the placenta and amygadala [2], [22]–[24]. The opposite effect of GR in these cells may rather relate to differential presence of transcription factors or coactivators such as SRC1a [18], [25]. One principle difference in cellular context between CRH containing cells in PVN and other tissues is that activation of the CRH gene in the paraventricular cells often will be accompanied by increased activation of the HPA axis, causing a quick rise in glucorticoid levels and GR activation. However, current data should be interpreted in the context of regulation of the CRH-promoter in the PVN, and do not give insights in the mechanisms governing the cell-specific effects of glucocorticoids on CRH expression.

It is well known that acute exogenous steroid treatment effectively suppresses stress-induced expression of CRH mRNA in rats [26]. However, the current study using a model system shows that repression is markedly attenuated if GR activation is initiated with as little as a 10 minutes delay. The critical time-window for effective repression by glucocorticoids may have interesting implications in the control of CRH expression *in vivo.* The order of activation of both signaling pathways is variable, and depends on the history of stress and glucocorticoid exposure, as well as the circadian and ultradian pulsatility of glucocorticoid levels [27]. Therefore, it is likely that effective GR-mediated repression of the stress-induced CRH mRNA expression will only occur in specific situations. We conclude that the differences in timing of stimulatory and repression signals are of consequence for adaptation of the organism to stress.

## Materials and Methods

### Reporter assays

0.1×10^6^ cells were transiently transfected in 24-wells plate using Lipofectamine 2000 (Invitrogen, Breda, The Netherlands) according to the manufacturer's instructions. Per well, 200 ng of the hCRH-luc reporter plasmid [18] or the 3×GRE containing TAT3-luc reporter were transfected. The day after transfection, the cells were treated with 10 μM forskolin (Calbiochem, Darmstadt, Germany) and/or 0.1 μM of the synthetic glucocorticoid dexamethasone (DEX) and assayed for luciferase activity.

Experiments were performed at 4 wells per condition, and were repeated at least three times. Statistical analysis was performed using one way analysis of variance (ANOVA) and statistical significance (*) was determined with Tukey's multiple comparison tests with p<0.05.

### Immunofluorescent staining of the GR

A day prior stimulation, 30×10^3^ AtT-20 cells were grown in chamber slides. Following stimulation, cells were fixed in 4% paraformaldehyde, permeabilized with Triton X-100 and blocked with 5% normal goat serum. Cells were incubated with a GR-specific antibody (M20; dilution 1∶500; Santa Cruz biotechnologies) during 60 minutes, washed and subsequently incubated for 60 minutes with a secondary goat anti-rabbit Alexa Fluor 488 antibody (dilution 1∶750; Invitrogen, Breda, The Netherlands). After incubation, cells were washed and counterstained for 10 min with Hoechst 33528. All sections were embedded in polyaquamount (Polysciences, Inc.) and visualized with an immunofluorescence microscope (Leica DM6000). Control cells were incubated with equal amounts of non-immune rabbit serum (Santa Cruz), which was used as substitute for the primary antibodies. Nuclear immunoreactivity was measured using ImageJ 1.32j software (NIH, USA).
